# A Study on the Effectiveness of Rumor Control *via* Social Media Networks to Alleviate Public Panic About COVID-19

**DOI:** 10.3389/fpubh.2022.765581

**Published:** 2022-05-13

**Authors:** Ben Lu, Jinlu Sun, Bo Chen, Qi Wang, Qi Tan

**Affiliations:** ^1^School of Economics and Management, University of Chinese Academy of Sciences, Beijing, China; ^2^School of Humanities and Social Sciences, Beihang University, Beijing, China; ^3^Institute for Finance and Economics, Central University of Finance and Economics, Beijing, China; ^4^College of Liberal Arts and Sciences, National University of Defense Technology, Changsha, China

**Keywords:** COVID-19, behavior, social network, Weibo, rumor control

## Abstract

The COVID-19 outbreak triggered a massive spread of unverified news on social media and has become a source of rumors. This paper studies the impact of a virtual rumor control center (RCC) on Weibo user behavior. The collected COVID-19 breaking news stories were divided into positive, negative, and neutral categories, while the moderating effect model was used to analyze the influence of anti-rumor on user behavior (forwarding, liking, and commenting). Our research found that rumor refuting does not directly affect user behavior but does have an indirect moderating effect. Rumor refuting has a profound impact on user forwarding behavior in cases of positive and negative news. Specifically, when the epidemic becomes more serious, the role of rumor refuting becomes critical, and vice versa. Refuting rumors reduces user willingness to forward positive or negative news, with more impact on negative news. Time lag analysis shows a significant moderation of unverified news within 72 h of refuting rumors but indicated an apparent weakening trend over time. Furthermore, we discovered non-linear feature and counter-cyclical phenomena in the moderating effect of rumor refutation.

## Introduction

The COVID-19 pandemic has exerted unprecedented and devastating effects on human societies. Unlike previous pandemics such as the H1N1 flu in 1918, COVID-19 is spreading rapidly through an interconnected world. As countries adopt strict physical distancing and other measures to control the virus, people increasingly depend on global digital social media networks such as Facebook, Twitter, and Weibo. These platforms help users sustain contact with others, enhance interpersonal interactions, and share virus-related information. However, these digital social networks also serve to promote a different type of virus, misinformation. The viral dissemination of inaccurate scientific information *via* digital social media may be used as a political weapon to destroy public trust in governments (1, 2). The WHO uses the term “infodemic” for the wide distribution of large amounts of misinformation through social networks (3, 4). Infodemics must be controlled because of the potential harm they inflict on human societies. Some administrations have sought to limit COVID-19-related misinformation dissemination on social media by pressuring corporations such as Facebook, Weibo, and Twitter to take appropriate actions (5). Social media has enormous power to manage rumors and is considered a potential rumor control center (RCC).

COVID-19 has been a continuous hot topic on social networks since early 2020, with massive news generated every moment. An information cascade begins when a user asserts news in a tweet (6). RCC is an authority whose goal is to minimize the spread of fake news by generating a positive cascade (7). However, fake news is not simply false information. It may also be polarized content, satire, misreporting, commentary, persuasive information, and citizen journalism (8). In most instances, the sharer either does not know or does not suspect the news they are sharing is fake. Because sharing fake news may negatively impact relationships, in the presence of RCC, users may be more inclined to share positive information (9). McIntyre and Gibson (10) found that valence plays a significant role in readers' affect, in that positive news makes readers feel good. In theory, positive news has a beneficial effect on restraining the speed of spreading rumors (11), but a lack of empirical analysis exists on the role of RCC in this process. Unlike previous research that focused on true or false news, this study looks at the impact of RCC on social network user behavior from the perspective of positive and negative information to help us understand how RCC works. Classification of news according to positive, negative, and neutral allows us to provide a new path for the design of RCC strategies. Existing research often aims at the short-term impact of a single or separate news cascade. Fortunately, COVID-19, as a continuous event with a series of information cascades, creates a natural experiment for us to study the mid-to-long-term impacts of RCC on user social network behavior. This article examines how RCC changes user behavior from the vantage of positive, negative, and neutral news.

This study first collected breaking news data and rumor-refuting information on social media during the first round of COVID-19 outbreak in China in 2020, then classified them according to the valency of news, and used regression models to analyze the impact of rumor-refuting on user behavior. The marginal contribution of this paper is to analyze the impact of RCC on the spread of rumors in the early stage of COVID-19, which provides valuable insights for improving RCC's strategies in responding to sudden disease disasters. Further, this paper studies a series of epidemic news events, which can reveal the law of RCC's effect on rumors more completely than only focusing on the impact of a single event. The research results explain how RCC changes the news cascade and provides guidance for designing social media anti-rumor strategies. Rumors may be real or fake news, but since this article is based on the RCC perspective, it needs to be clarified that the rumor mentioned in this article refers to fake news.

### Hypothesis Development

Online media is the site of information propagation and the persistent discussions surrounding such information. When an individual receives news about COVID-19, for instance a rumor, he may turn to other sources to understand, evaluate, debunk, or verify the information, often depending on their prior beliefs (12). Users will also use RCC as an important source for assessing the credibility of the information. There are currently two ways to control fake news on social networks: one is to tag misinformation so that users can identify suspicious information (13, 14), the way Twitter does; the other is to continuously broadcast rumor-defending information through RCC accounts, such as Weibo. Both approaches have benefits, and glaring limitations. The first approach allows users to see the suspicious information tag, but flaws in the algorithm may miss some potential rumor seeds, such as puns or ironic expressions. The second approach uses an “anti-rumor” process, akin to the way rumors are spread (15, 16), but this process has a lag effect and uncertainty. Existing studies on the effectiveness of rebuttals have reached mixed findings. Some studies showed that rebuttals help reduce belief in rumors (17–19), while other studies revealed opposite results. For instance, there is the “backfire effect” where corrections actually increase the belief in rumors (20). Opposing views on the role of RCC may signify undiscovered mechanisms.

Like Twitter, Weibo is a platform for users to share, distribute, and obtain information based on their associations. Users can receive all information about COVID-19, including official announcements, news, rumors, and anti-rumors. Weibo publishes relevant messages related to COVID-19 in real-time in a prominent location, informing users of details such as the current number of infections and deaths, etc. The platform also established an official rumor-defying account to control the spread of rumors (Weibo RCC). As of 26 July 2020, this account had about 2.33 million registered followers and a total of 9,607 messages.

User behaviors on Weibo include clicking, forwarding, liking, and commenting. Clicking signifies user interest, forwarding represents user action to disseminate information, liking represents positive user attitude, and commenting indicates user interest in public discourse on a topic. Weibo builds a real-time Hot Topic Ranking (HTR) list based on the above data and makes recommendations on the user's homepage. The HTR is a structured news cascade, composed of the 50 most popular news at the time. After clicking on one of the news items, users see a summary and the most popular user comments right below it. Although HTR uses an objective way to describe news, the news itself may be positive, negative, or neural, which is a crucial factor affecting user behavior (9, 11).

The definition of positive and negative news is the basis of this research. Harcup and O'Neill (21) defined good news as “stories with particularly positive overtones such as rescues and cures” and bad news as “stories with particularly negative overtones, such as conflict or tragedy.” McIntyre and Gibson (10) defined a positive news as one that focuses on the benefits of an event or issue and a negative news as one that focuses on the harmful outcomes of an event or issue. The definition of positive and negative news in this study is based on the previous research and the characteristics of COVID-19 news. Positive news is good for building public confidence in the fight against the epidemic. These news include posts on medical staff actively treating patients, online charity concerts held by celebrities, public donations of medical supplies, and signs of improvement in the epidemic, such as zero new confirmed cases in a region, reopening of closed roads, or active development of a new vaccine. Negative news, on the other hand, can harm public sentiment. These posts include government announcements of city closures and delays in the opening of schools. News items that were neither positive nor negative were collectively classified as neutral.

There is a growing body of work suggesting that responses to positive and negative information are asymmetric—that negative information has a much greater impact on individuals' attitudes than does positive information (22). Scholars who focus on information diffusion have suggested that people might be more likely to share positive rather than negative messages in an effort to signal their identity or enhance their self-presentation (23, 24). In contrast to their findings, Hansen et al. (25) found that negative news messages were shared more than positive news messages on Twitter. Soroka and McAdams (26) conducted a psychophysiological experiment showing that negative news elicits stronger and more sustained reactions than does positive news. When the news content is negative, it produces a stronger reaction and/or higher attention, which may be due to the framing effect caused by the mediating role of user emotions (27). Emotion is the regulator of people's social behavior (28), and the content characteristics of social media will be regulated by emotions and affect people's engagement (29), and even trigger aggressive behaviors (30). In the face of disease risk, it has also been proven that emotional variables such as fear and anger can generate positive preventive behaviors through the use of social media (31). In the process of dispelling rumors, users' social media behaviors will probably be affected by the valence of news. In order to quantify this impact, this article uses the frequency of rumor refuting to measure the intensity of rumor refuting, and proposes the following hypotheses:

*H1*. The intensity of anti-rumor affects users' behaviors with different impacts on positive, negative and neutral news.

*H2*. The intensity of anti-rumor plays a moderating role between public panic and user behaviors with different impacts on positive, negative and neutral news.

## Methods

### Model Setting

A linear regression model was used for empirical analysis, where the dependent variable was the user social behavior on COVID-19 news. The online user behavior regarding COVID-19 information resulted from the combined effects of receiving varied information during the study. Therefore, the data related to user behavior can effectively measure the public response to rumor rebuttals. This study collected public comments on Weibo as the basis of the analysis, but no patient and public participated in the experiment.

The social media panic is closely related to the spread of the pandemic and media reports (32). Therefore, we used the reported incidence of infections to measure public panic. Furthermore, the peak time of the epidemic (5 February 2020) also exerted a powerful impact on public panic; thus, a peak dummy variable was introduced.

To test H1, the main effect model is as follows:


$$\begin{array}{l} behavior_{t}=\alpha_{0}+\;\alpha_{1}\times {anti\text{\_}rumor}_{t}+\alpha_{2}\times panic_{t}+\alpha_{3}\times \\ \;{peak\text{\_}dum}_{t}+\varepsilon_{t} \end{array}$$

To test H2, the moderating effect model was postulated in the following manner:


$$\begin{array}{l} behavior_{t+T}=\alpha_{0}+\;\alpha_{1}\times {anti\text{\_}rumor}_{t}+\alpha_{2}\times panic_{t}+\alpha_{3}\times \\ \;{peak\text{\_}dum}_{t}+\alpha_{4}\times panic_{t}\times {anti\text{\_}rumor}_{t}+\varepsilon_{t} \end{array}$$

where *behavior*_*t*_ represents user behavior, *anti*_*rumor*_*t*_ and *panic*_*t*_ represents the intensity of rumor rebuttals and the degree of public panic, respectively. The dummy variable *peak*_*dum*_*t*_ represents the epidemic peak (before or after the peak). α_0_, α_1_, α_2_, α_3_, α_4_ are coefficients. *T* reflects lag time and *panic*_*t*_ × *anti*_*rumor*_*t*_ represents the interaction effect.

### Data

This study collected epidemic-related data on Weibo, China's largest social networking platform, from 1 January 2020 to 31 March 2020, including 4,004 COVID-19-related news and 1,150 RCC anti-rumor information. The daily number of infections comes from official disclosures. Data collection date is 23 April 2020. COVID-19 news items were filtered and classified manually into three categories according to content attributes: positive, negative, or neutral. Furthermore, since the collected data were cross–sectional, we restructured it by the hour according to the “48-h allocation” method described below. Finally, 1,657 time-series samples were obtained. The calculation method of each variable is outlined below:

*behavior*_*t*_: Around 4,004 news items on COVID-19 were obtained after manual screening, and the number of clicks, forwards, likes, and comments of each post were also collated. The calculation was accomplished by counting the number of clicks, forwards, and comments according to the hour. We subsequently computed the value of forwards/clicks, likes/clicks, comments/clicks for every hour to use as dependent variables. Finally, *behavior*_*t*_ was recalculated according to a “48-h allocation” approach.

*anti*_*rumor*_*t*_: The data were extracted from Weibo's RCC and yielded a total of 1,150 records of effective anti-rumors. The rumor refutation was carried out by Weibo RCC at different times every day, and we counted its release frequency every hour. The number of RCC releases per hour was used as an indicator of rumor refutation intensity after being processed through the “48-h allocation.”

*panic*_*t*_: China's official daily release of newly confirmed COVID-19 cases (*confirm_add*), fresh suspected infections (*suspect_add*), and current COVID-19 related deaths (*dead_add*) were compiled to measure public panic. Since the number of suspected cases had a great impact on the Chinese public in the early stage of the epidemic, the model used *suspect_add* as the main indicator. For the sake of robustness, we used *confirm_add* and *dead_add* as alternative indicators (see Supplementary Material).

*panic*_*dummy*_*t*_: Public panic may differ significantly before and after the peak of the pandemic. Therefore, it was recorded as 0 before 5 February 2020, and as 1 after that date.

**Figure 1:**
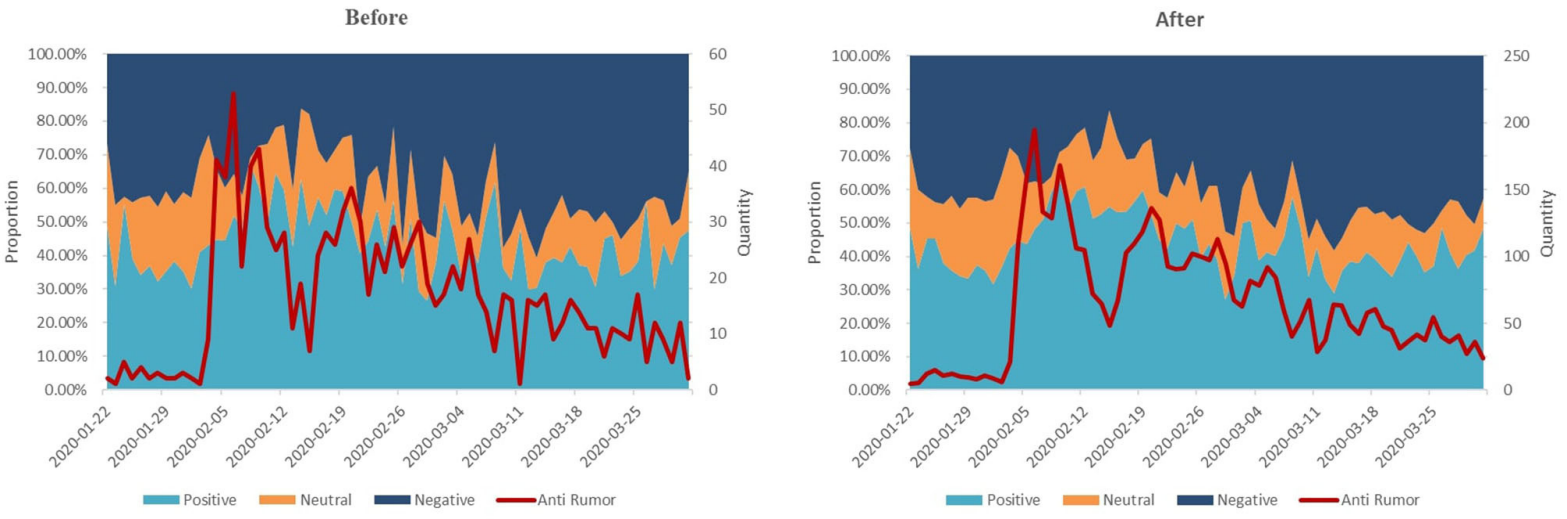
Daily accumulation graph of positive, negative, and neutral news and the number of anti-rumors before (left) and after (right) the “48-h allocation.” The primary vertical axis represents the proportion of positive, neutral and negative rumors, while the secondary vertical axis represents the strength of the rumor-refuting information.

*Forty-eight-hour allocation*: The power of information dissemination on social networks shows the characteristics of non-linear decline. According to Kwak et al. (33) on the spread of Twitter information, more than 50% of the forwarding behavior occurred within 1 h of the posting an item, and more than 75% of the forwarding behavior occurred within 1 day. Based on the above research, we used the weight allocation method to simulate the characteristics of social network information dissemination within 48 h, and its weight will continue to decrease over time, which is the so-called “48-h allocation.” Then, *behavior*_*t*_ and *ant*_*i*_*r*_*umort*_ were processed according to the “48-h allocation.” The specific processing method entailed starting from the release time of the entry. 50% weight was allocated for the first hour, 25%/23 for the following 23 h, and 25%/24 for the next 24 h. For example, if an entry was listed at 0:00 on 1 January 2020, it was recorded as 50% × (number of clicks, forwards, comments, likes) from 0:00 to 1:00 on 1 January 2020, as 25%/23 × (number of clicks, forwards, comments, likes) from 1:00 to 23:00, and as 25% /24 × (number of clicks, forwards, comments, likes) from 0:00 to 23:00 on 2 January 2020. Table 1 outlines the variable definitions. The statistical description and correlation coefficient after data processing are exhibited in Table 1. Figure 1 shows the daily accumulation of positive, negative, and neutral news and the number of anti-rumors before (left) and after (right) the “48-h allocation.”

**Table 1:** Variable definitions and statistical description.

**Variables**	**Definition**	**Indicators**	**Positive**					**Neutral**					**Negative**				
			***N***	**Mean**	**Sd**	**Min**	**Max**	***N***	**Mean**	**Sd**	**Min**	**Max**	***N***	**Mean**	**Sd**	**Min**	**Max**
*behavior_*t*_*	Forwards/hits	Forward_d _clicks	1,657	−5.079	0.773	−9.781	−1.221	1,640	−4.952	0.972	−11.84	−2.249	1,657	−5.381	0.841	−9.216	2.112
	Likes/hits	Like_d_clicks	1,657	−1.384	0.704	−6.072	1.649	1,640	−1.312	0.897	−8.167	2.381	1,657	−1.534	0.663	−6.247	1.39
	Comments/hits	Comment_d _clicks	1,657	−4.811	0.604	−8.833	−1.973	1,640	−4.592	0.815	−12.02	−1.85	1,657	−4.778	0.588	−9.204	−2.344
	New confirmed daily	Confirm_add	1,657	5.655	1.933	2.079	9.626	1,640	5.669	1.935	2.079	9.626	1,657	5.655	1.933	2.079	9.626
*panic_*t*_*	New deaths daily	Dead_add	1,657	3.317	1.198	0	5.537	1,640	3.336	1.182	0	5.537	1,657	3.317	1.198	0	5.537
	New suspected daily	Suspect_add	1,657	5.848	1.988	2.833	8.581	1,640	5.865	1.988	2.833	8.581	1,657	5.848	1.988	2.833	8.581
*anti*_*rumor*_*t*_	Number of anti–rumors released by RCC	Anti_rumor	1,657	−1.186	1.495	−4.522	1.738	1,640	−1.175	1.493	−4.522	1.738	1,657	−1.186	1.495	−4.522	1.738
*peak*_*dummy*_*t*_	Peak day	Peak_dum	1,657	0.809	0.393	0	1	1,640	0.807	0.395	0	1	1,657	0.809	0.393	0	1

## Results

### Main Effect and Moderating Effect

According to model (1), the main effect regression was performed on positive news, negative news, and neutral news, with *behavior*_*t*_ as the dependent variable, and *anti*_*rumor*_*t*_, *panic*_*t*_, and *panic*_*dum*_*t*_ as explanatory variables. Then, according to the model (2), the moderating effect variable *panic*_*t*_ × *anti*_*rumor*_*t*_ was added, and finally, 18 regression models were established. To reduce collinearity, *panic*_*t*_, *and anti*_*rumor*_*t*_ are mean-centered. The regression results show that rumor refuting has a complex impact on user behavior with different news types (as shown in the Table 2).

**Table 2:** Main effect and moderating effect regressions.

	**Positive**						**Negative**						**Neutral**					
**Variables**	**Forward_d_clicks**		**Like_d_clicks**		**Comment_d_clicks**		**Forward_d_clicks**		**Like_d_clicks**		**Comment_d_clicks**		**Forward_d_clicks**		**Like_d_clicks**		**Comment_d_clicks**	
Suspect_add	0.0251* (2.33)	0.0187 (1.73)	0.0268** (2.65)	0.0286** (2.81)	0.0600*** (6.95)	0.0608*** (6.99)	0.0273* (2.27)	0.0161 (1.35)	0.0117 (1.20)	0.0121 (1.23)	0.0551*** (6.45)	0.0551*** (6.40)	0.137*** (10.63)	0.140*** (10.73)	−0.00435 (−0.35)	−0.00395 (−0.31)	0.0681*** (5.87)	0.0733*** (6.30)
Peak_dum	−0.596*** (−8.82)	−0.719*** (−10.01)	−0.347*** (−5.47)	−0.312*** (−4.60)	−0.163** (−3.02)	−0.148* (−2.56)	−0.347*** (−4.60)	−0.562*** (−7.08)	−0.0992 (−1.62)	−0.0908 (−1.38)	0.0092 (0.17)	0.00892 (0.16)	−0.636*** (−7.82)	−0.590*** (−6.80)	−0.820*** (−10.34)	−0.812*** (−9.59)	−0.371*** (−5.09)	−0.269*** (−3.47)
Anti_rumor	−0.00276 (−0.17)	−0.00592 (−0.37)	−0.0127 (−0.84)	−0.0118 (−0.78)	−0.0079 (−0.62)	−0.00751 (−0.58)	−0.0551** (−3.07)	−0.0606*** (−3.43)	−0.0218 (−1.50)	−0.0216 (−1.49)	−0.0309* (−2.43)	−0.0309* (−2.43)	0.0294 (1.52)	0.0309 (1.59)	0.0372* (1.96)	0.0374* (1.97)	0.0137 (0.78)	0.017 (0.98)
Suspect_add _anti_rumor		0.0320*** (4.80)		−0.00903 (−1.43)		−0.00398 (−0.74)		0.0559*** (7.57)		−0.00218 (−0.36)		0.0000718 (0.01)		−0.0122 (−1.50)		−0.00211 (−0.27)		−0.0274*** (−3.78)
_cons	−4.747*** (−40.49)	−4.499*** (−74.16)	−1.275*** (−11.61)	−1.131*** (−19.77)	−5.039*** (−53.68)	−4.691*** (−95.92)	−5.326*** (−40.66)	−4.930*** (−73.49)	−1.548*** (−14.57)	−1.460*** (−26.38)	−5.145*** (−55.39)	−4.786*** (−98.88)	−5.210*** (−37.03)	−4.475*** (−61.17)	−0.581*** (−4.24)	−0.657*** (−9.20)	−4.676*** (−37.06)	−4.374*** (−66.91)
*N*	1,657	1,657	1,657	1,657	1,657	1,657	1,657	1,657	1,657	1,657	1,657	1,657	1,640	1,640	1,640	1,640	1,640	1,640
*R* ^2^	0.115	0.127	0.063	0.064	0.071	0.071	0.067	0.099	0.012	0.012	0.039	0.039	0.196	0.197	0.104	0.104	0.081	0.089
adj. *R*^2^	0.113	0.125	0.061	0.061	0.069	0.069	0.066	0.096	0.01	0.01	0.037	0.036	0.195	0.196	0.103	0.102	0.079	0.087
F	71.38	60	36.75	28.09	42.1	31.7	39.84	45.2	6.809	5.136	22.18	16.63	133.3	100.6	63.42	47.56	48.03	39.88
df_m	3	4	3	4	3	4	3	4	3	4	3	4	3	4	3	4	3	4
df_r	1,653	1,652	1,653	1,652	1,653	1,652	1,653	1,652	1,653	1,652	1,653	1,652	1,636	1,635	1,636	1,635	1,636	1,635

*t statistics in parentheses*.

*p < 0.05, **p < 0.01, ***p < 0.001.

### Positive News

**Figure 2:**
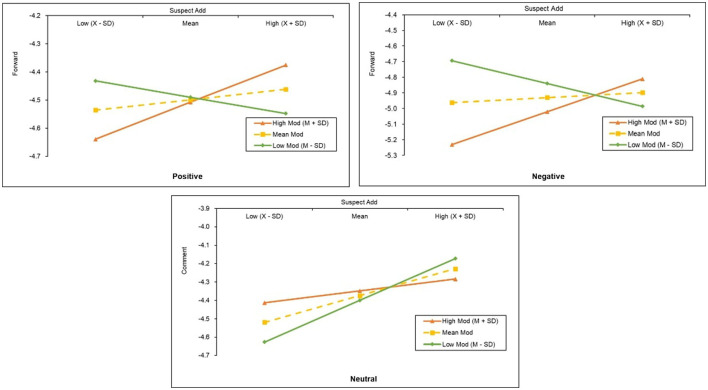
Simple slope test graphs. A simple slope plot represents the direction and strength of the moderating effect.

The number of new suspected cases (*suspect_add*) significantly affected user forwarding and commenting behavior *via* moderating effect. Before the moderating effect was added, the intensity of rumor rejection did not affect the spread of positive news, rejecting H1). However, there was an interaction effect between the anti-rumor intensity (*anti_rumor*) and the number of new suspected cases (*suspect_add*) (β = 0.0320, *p* < 0.001), and H2 cannot be rejected. β is the non–standard coefficient. The simple slope graph shows that with the increase of new suspected cases, refuting rumors stimulated user enthusiasm for forwarding positive news (Figure 2). The dummy variable peak epidemic time (*peak_dum*) was negatively significant in all regressions, proving the impact of the epidemic peak time on user behavior. After the peak of the epidemic, there was a decline in reposting, liking, and commenting on positive news.

### Negative News

The number of new suspected cases (*suspect_add*) also significantly affected user forwarding and commenting behavior *via* both main and moderating effect. Anti-rumors can significantly inhibit the spread of negative news: the regression coefficients of the variable *anti_rumor* for forwarding (*forward_d_clicks*) and comments (*comment_d_clicks*) were (β = −0.0606, *p* < 0.001) and (β = −0.0309, *p* < 0.05), respectively, and H1 cannot be rejected. Furthermore, the model also had a significant moderating effect on forwarding behavior, consistent with the positive news results. However, the dummy variable *peak_dum* was only negatively significant for the forwarding behavior. This shows that, compared to positive and neutral news, users still maintained strong enthusiasm for negative news even after the peak.

### Neutral News

The number of new suspected cases (*suspect_add*) will also significantly affect user forwarding and commenting behavior through only moderating effect. Before we added the moderation effect, the intensity of rumor rejection had no significant impact on the forwarding and comments on positive news. Although it was significant for liking (β = 0.0374, *p* < 0.05), it became insignificant after the moderation effect was added. The moderating effect for commenting is significant (β = −0.0274, *p* < 0.001).

To further analyze the meaning of the moderating effect, a simple slope test was conducted (34) (see the Figure 2). Figure 2 shows that varying intensities of anti-rumor exercised significant differences in the moderating effect of public behavior (high, median, and low mode represents high, middle and low level, respectively). For positive and negative news, the moderating effect had a non–linear feature. When the intensity of anti-rumor was higher than a certain critical value, the greater the intensity, the stronger the influence on user behavior, and vice versa. There was no similar rule for neutral news, but as the intensity of rumor refuting increased, user enthusiasm for neutral news declined precipitously.

### Hysteresis Analysis

**Figure 3:**
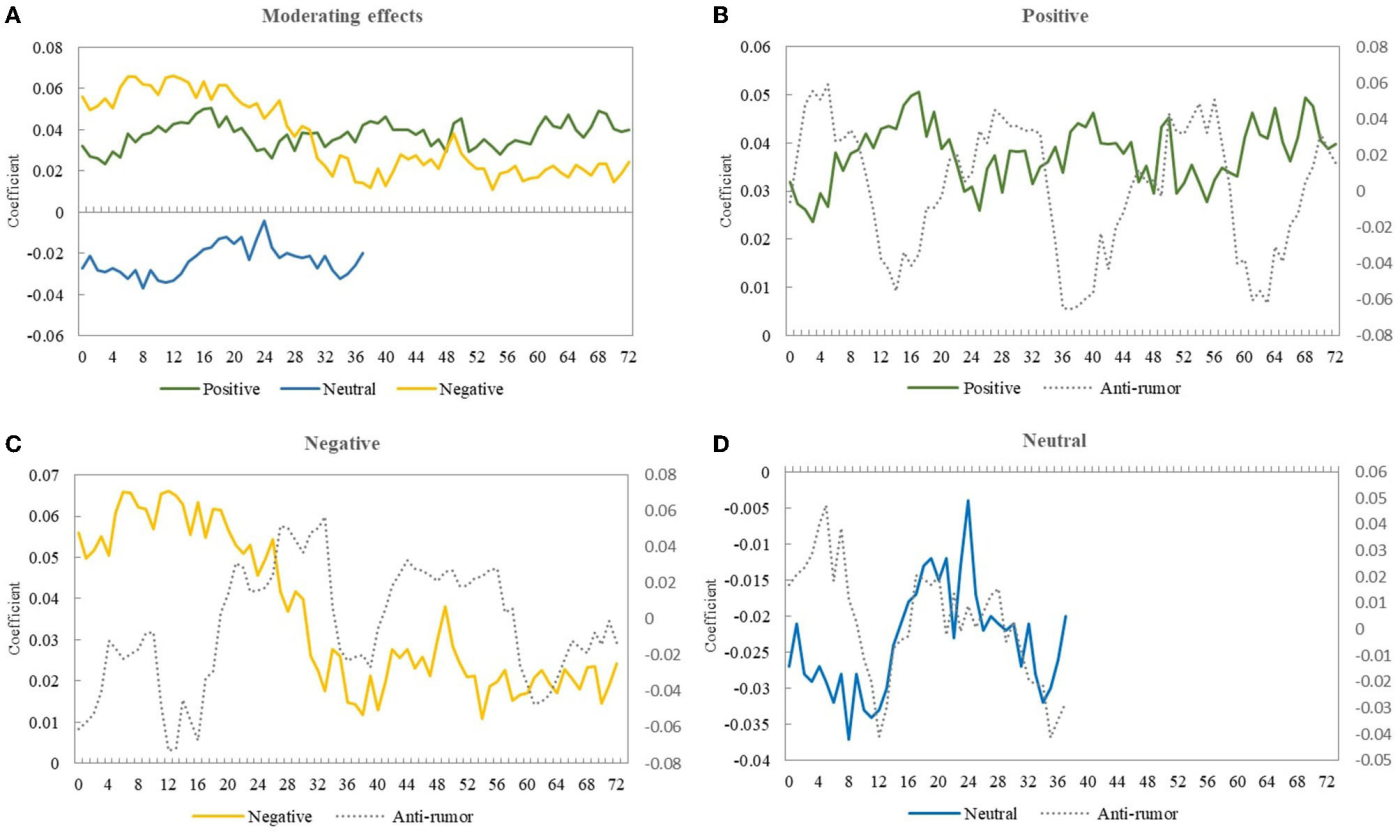
The moderating effect of rumor refutation over time. **(A)** Represents the trend of the moderating effect of positive, neutral and negative information over time. **(B–D)** Represent the comparison of the three types of information and the corresponding moderating effects of refuting rumors.

The dissemination of rumor refutation information takes a certain amount of time; thus, a lag effect may exist. Therefore, the explanatory variable *behavior*_*t*+*T*_ was used as a lag item to test the moderating effects of anti- rumor messages released within 72 h. The results evinced the significant moderating effect of rumor refutation within 72 h but indicated an apparent weakening trend over time (see the Figure 3A). Specifically, the impact of rumor rebuttal on negative news peaked within 12 h and then continued to decline until reduced to half after 32 h. The moderating effect of positive news remained consistent within a relatively stable range and fluctuated. For neutral news, the mediating effect was no longer significant after 36 h.

After further analyzing the change trend of the coefficients of anti-rumor and moderating effect, the results revealed a clear counter-cyclical relationship between them, which was especially obvious for positive news (see the Figures 3B–D). When the anti-rumor effect decreased, the moderating effect began to rise. A reasonable explanation for this phenomenon is that the role of anti-rumor has a certain lead time. After it reaches a peak, the moderating effect starts to work, and the lead time is estimated at 16–20 h.

## Discussion

This study reveals the complicated mechanism of rumor refuting on user behavior using RCC broadcast methods. Refuting rumors does not directly affect user behavior, but indirectly changes it through a moderating effect. For both positive and negative news, rumor refuting has a positive impact on user forwarding behavior through interactive effects. When the epidemic becomes more serious, the role of rumor refuting intensifies, and vice versa. Further, there is a counter-cyclical phenomenon between the main effect of refuting rumors and the moderating effect. When the main effect begins to weaken, the moderating effect increases instead. This shows that RCC directly affects the spread of rumors first, and then further affects the wider social behavior of users. This shows that refuting rumors can not only reduce the spread of rumors, but also affect users' reactions to negative news more widely, which further reduces the environment for the spread of rumors. This finding has not received sufficient attention in past research. As far as neutral information is concerned, a fascinating discovery has emerged from our data. We found that dispelling rumors stimulates users to be more expressive and opinionated as the epidemic worsened. This finding indicates that neutral news is more likely to originate rumors, because users interpret uncertainty in a variety of ways, often promoting the appearance of hearsay.

From the vantage of user behavior, rumor refuting reduces user willingness to forward positive and negative news simultaneously, with a deeper impact on negative news, which is consistent with previous researches (23, 24). However, refuting rumors will not alter user liking and commenting behavior, proving that RCC only impacts the spread of information but hardly affects user enthusiasm for participating in discussions. Further examination is needed of the non–linear feature in the mediation effect of rumor refuting. If it exists, greater flexibility is necessary to design rumor-refuting strategies. In any case, categorizing news into positive, negative, and neutral and then formulating targeted strategies to dispel rumors can effectively improve the efficiency of RCC. It is much cheaper to classify news in advance than to identify rumors after the fact. Furthermore, the peak time of the epidemic was found to exercise a significant impact on user behavior. After the epidemic peaked, user enthusiasm for all types of news dropped significantly. These findings suggest that RCC can break the framing effect produced by public sentiment (27), thereby alleviating public panic caused by COVID-19, but it requires sophisticated intervention strategies.

The main contribution of this study is to find that RCC can not only suppress the spread of rumors, but also can further affect the wider behavior of users, thereby helping to dispel rumors. This finding helps to optimize the design of RCC strategies. For example, targeting technology can be used to broadcast rumor-refuting information to specific groups of people based on the valence of news. But the research also has some limitations. First, its conclusions are limited and applicable to China's cultural environment because Weibo data were used for the investigation. Therefore, it is necessary to conduct a cross-cultural comparative study of user behavior. Second, the timing of this study was limited to the outbreak stage of the epidemic in China. However, the global transmission characteristics of COVID-19 have undergone significant changes, and the user psychology may have changed. Finally, this study uses behavioral data for correlation analysis, which cannot fully reveal the operating mechanism of RCC.

## Data Availability Statement

The original contributions presented in the study are included in the article/Supplementary Material, further inquiries can be directed to the corresponding authors.

## Ethics Statement

The studies involving human participants were reviewed and approved by Central University of Finance and Economics. Written informed consent for participation was not required for this study in accordance with the national legislation and the institutional requirements.

## Author Contributions

BL: writing and editing of the paper. JS and BC: literature review and methodological design. QW: data collection and processing. QT: discussion part of the paper. All authors contributed to the article and approved the submitted version.

## Funding

This paper is supported by the National Social Science Foundation (Project Number 19BGL300).

## Conflict of Interest

The authors declare that the research was conducted in the absence of any commercial or financial relationships that could be construed as a potential conflict of interest.

## Publisher's Note

All claims expressed in this article are solely those of the authors and do not necessarily represent those of their affiliated organizations, or those of the publisher, the editors and the reviewers. Any product that may be evaluated in this article, or claim that may be made by its manufacturer, is not guaranteed or endorsed by the publisher.
